# Quantitative analysis of the trajectory of simulated basilar apex aneurysms through the internal carotid artery to assess the need for an orbitozygomatic approach

**DOI:** 10.1007/s00701-016-3018-7

**Published:** 2016-11-15

**Authors:** Yasushi Motoyama, Yasuo Hironaka, Fumihiko Nishimura, Pritam Gurung, Ryota Sasaki, Yasuhiro Takeshima, Ryosuke Matsuda, Kentaro Tamura, Ichiro Nakagawa, Young-Su Park, Hiroyuki Nakase

**Affiliations:** 1 Department of Neurosurgery, Nara Medical University, 840 Shijo-cho, Kashihara, Nara 634-8522 Japan; 2 Ohnishi Neurological Center, Akashi, Hyogo Japan

**Keywords:** 3DCTA, Basilar apex aneurysm, Orbitozygomatic approach, Internal carotid artery, 3D simulation

## Abstract

**Background:**

The aim of this study was to identify the correlation between the location of the internal carotid artery (ICA) and the need for an orbitozygomatic approach (OZA) when approaching a basilar apex (BX) aneurysm.

**Materials and methods:**

By imaging the virtual trajectory to access the basilar artery (BA) through the ICA, the correlations among the height of the BX, the height and lateral breadth of the bifurcation of the ICA, and the need for removal of the orbital rim or zygomatic arch were investigated using three-dimensional computed tomography angiography (3DCTA) data of approximately 80 random samples not limited to BX aneurysms. Furthermore, the utility of 3D simulation to determine the need for the OZA was verified using data from five patients with BX aneurysms.

**Results:**

The height of the bifurcation of the ICA was inversely correlated and the height of the BX was positively correlated with the need for the OZA (both *p <* 0.017). Among patients undergoing surgery, clipping was successfully performed without the OZA in two patients in whom the distance from the simulated skull point on the extended line from the BX through the bifurcation of the ICA was more than 4 cm from the zygoma and orbital rim.

**Conclusions:**

It is necessary to determine the spatial relationship between the basilar artery and the ICA to decide whether the OZA is needed for surgery. Correlations of the height of the ICA and BX with the need for the OZA were not very strong individually, though they were significant. Therefore, simulation using 3DCTA appears to be important for planning the surgical approach for the treatment of BX aneurysms.

## Introduction

Basilar apex (BX) aneurysms, including basilar tip and basilar artery-superior cerebellar artery (BA-SCA) aneurysms, are difficult to treat because of their deep location. Although these aneurysms have been treated with endovascular coil embolization in many recent cases, some patients still require direct surgical intervention, including those with small aneurysms, who have a poor interventional access route, and those who have contraindications to receiving iodinated agents. Access routes to the interpeduncular or prepontine cistern for observation of the aneurysms include the pterional, subtemporal, and transpetrosal approaches; the specific approach is selected on the basis of aneurysm size, the projection of the dome, and the location of the neck of the aneurysm. The orbitozygomatic approach (OZA) has been useful in accessing BX aneurysms, especially in cases where it is in a high position, because this approach can facilitate upward and oblique viewing from below through the wide operative space [3, 8, 9, 16]. However, the OZA needs additional removal of the orbital rim and zygomatic arch, in addition to standard pterional craniotomy, which increases invasiveness, the risk of facial nerve palsy, temporal muscle atrophy, and deformity after surgery, and results in an extended operative time. Appropriate selection of the OZA requires indications that have yet to be established. The trajectory to BX aneurysms in the interpeduncular or prepontine cisterns has been suggested to be related to not only the height of the apex of the basilar artery (BA), but also the height and lateral breadth of the bifurcation of the internal carotid artery (ICA) [9]. To access BX aneurysms across the ICA, it is better to simulate how the ICA blocks the operative field to appropriately observe the target. The use of the three-dimensional (3D) relationship between the ICA and the BA to help select the approach for BX aneurysms has not been well studied. Therefore, we used data from three-dimensional computed tomography angiography (3DCTA) to construct a virtual image of the trajectory needed to obtain an appropriate operative field in which the BX aneurysm is well visualized through the ICA. According to this image, we investigated how the BA and ICA are related to the need for the OZA, which is divided into two components, the orbital rim and the zygomatic arch, when approaching BX aneurysms.

In addition, data from patients with BX aneurysms were reviewed to simulate their trajectory to access the BX through the ICA. Lastly, the utility of simulation using 3DCTA to select the approaches to BX aneurysms was validated.

## Materials and methods

### Study population and 3DCTA sampling

Eighty samples of 3DCTA from 40 patients were used to simulate access to a BX through the ICA. Ten patients from each decade of patient age between 30 and 70 years were chosen randomly from those who underwent 3DCTA at Nara Medical University between 2014 and 2015. The average patient age was 49.7 ± 11.0 years (range, 31–68 years). The patients included 19 females and 21 males. Indications for examination included a variety of cerebrovascular diseases, brain tumors, and other lesions. Exclusion criteria consisted of occlusive disease involving the ICA or BA, apparent space-occupying lesions affecting the location of the ICA and BA (such as paraclinoid tumors), intrinsic tumor, stroke, and traumatic lesions with marked brain shift. Patients with cerebrospinal fluid (CSF) leakage (e.g., those in the early postoperative period with potential intracranial hypotension) were excluded because of possible shift of the whole brain structure.

### CT protocol

A standard protocol was used to acquire 3DCTA images on a multidetector CT scanner (SOMATOM Definition Flash, Siemens Medical Solutions, Forchheim, Germany). A dual-energy protocol was used with tube A (140 kV, 70 mA) and tube B (80 kV, 297 mAs), with 2 × 32 × 0.6-mm collimation, pitch 0.8, and a rotation time of 0.5 s. A premonitoring standard brain scan without contrast was then performed to show the anatomic level (first cervical vertebra) for the start of contrast bolus tracking. After placement of an intravenous catheter in the patient’s antecubital vein, an 80-ml intravenous contrast bolus was injected at the rate of 4 ml/s. The monitoring scan was performed simultaneously. Then, scanning (0.6-mm collimator, 1.0-mm slice thickness, 0.5-mm interval) was initiated when the contrast was confirmed to have filled the cervical carotid arteries, in which the HU values reached 150.

## Workstation

Post-processing was performed on an Aquarius NET TM Workstation (TeraRecon, San Mateo, CA, USA). This software automatically provides 3D vessel images, as well as overlook, front, and rear views, together with a view of the skull base bony structure. In addition, three multiplanar reformation planes (sagittal, coronal, and axial) are shown for the location of the camera. Data obtained by scanning 3DCTA were immediately transferred to a computer server in our institution, and, within 5 min, the 3D images could be manipulated on any computer terminal, being always available for manipulation by surgeons using software for preoperative assessment.

### ABC line as a virtual trajectory

**Fig. 1:**
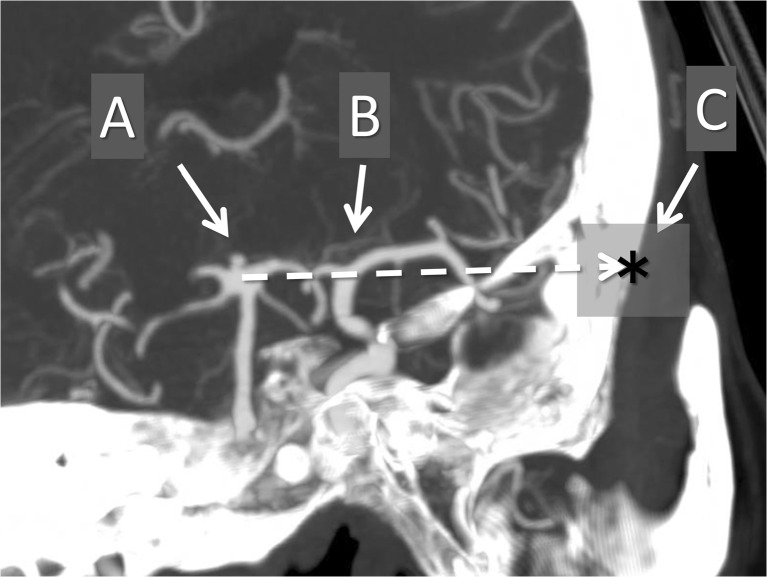
The apex of the BA that is set as point A is connected to the bifurcation of the ICA, which is set as point B, to make the line AB. The line connecting A and B is extended and projected onto the cranium, which is set as point C. The ABC line on the image is provided by maximum intensity projection (MIP)

We used 3D data from CT scans with contrast to construct a virtual image of the trajectory to approach BX aneurysms. The apex of the BA, set as point A, was connected with the bifurcation of the ICA, which was set as point B, to make the line AB. This line was the corridor made by 3D virtual images constructed on a workstation, and it reflected the real trajectory for visualizing the BX aneurysm through the ICA. The line connecting A and B was extended and projected onto the cranium, which was set as point C. During surgery, point C should be the center of the cranial window, if possible, and it represents the limit of the cranial window for observing the interpeduncular cistern through the ICA (Fig. 1).

**Fig. 2:**
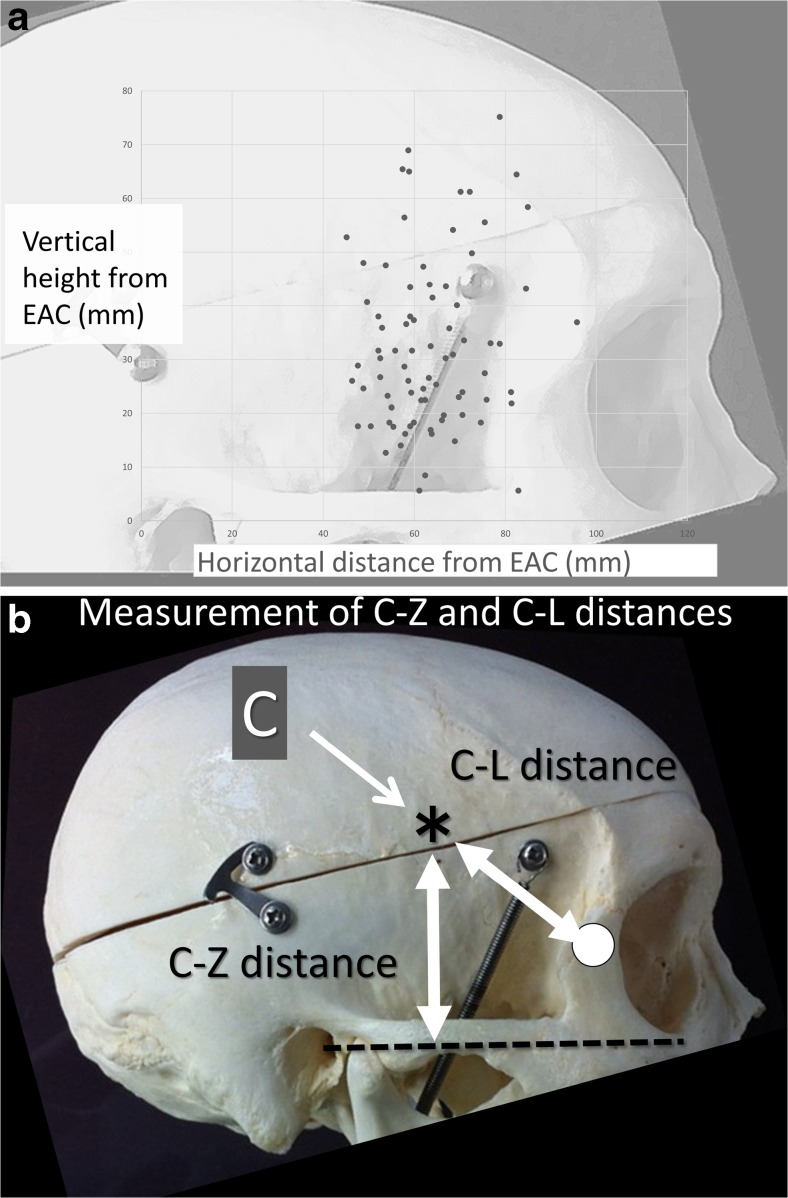
The coordinates of point C from the ipsilateral external auditory canal are calculated on a workstation and recorded. The distribution of point C for all patients is shown on the scatter diagram (**a**). The distance from point C (*asterisk*) to the zygomatic arch (*dotted line*), the C-Z distance, which inversely reflects the need for removal of the zygoma. The distance from point C to the lateral canthus (*white circle*), C-L distance, which inversely reflects the need for removal of the orbital rim (**b**)

The coordinates of point C from the ipsilateral external auditory canal were calculated on a workstation and recorded. The distribution of point C for all patients is shown by the scatter diagram (Fig. 2a).

## Measurement of points A, B, and C

We measured the following distances for evaluation of the need for the OZA for BX aneurysms through the ICA, which were calculated using measurement tools on the workstation (Figs. 1 and 2b).

1. The height of the apex of the BA from the dorsum sellae: height of A
2. The height of the bifurcation of the ICA from the dorsum sellae: height of B
3. The lateral breadth of the bifurcation of the ICA from the midline: width of B
4. The distance from point C to the zygomatic arch: C-Z distance (inversely reflects the need for removal of the zygoma)
5. The distance from point C to the lateral canthus: C-L distance (inversely reflects the need for removal of the orbital rim)

## Interpretation and statistical analysis

If point C is close to the zygomatic arch, zygotomy is necessary for the appropriate trajectory. If point C is near the lateral canthus, frontal orbitotomy is required. The distance between point C and the zygomatic arch (inversely related to the need for zygotomy) was measured on the workstation. The distance between point C and the lateral canthus (corresponds to the need for frontal orbitotomy) was calculated. We investigated the correlation between the C-Z and C-L distances with the height of A and the height and width of B. The patients were categorized according to age for sub-analyses. Measurements are expressed as means ± standard deviation (SD) or medians. The relationships between the distances from point C and measurements of points A and B obtained from the workstation were investigated using Pearson’s correlation. A P value of 0.05 or lower was considered to indicate significance. The Bonferroni method was used to correct for multiple comparisons as appropriate.

## Results

The mean vertical height and occipitofrontal distance from point C to the external auditory canal were 62.6 ± 10.1 mm (range, 4.2–76.7 mm) and 31.6 ± 16.3 mm (range, 45–95.7 mm), respectively. The scatter diagram shows a wide distribution of point C, which is located at least 4 cm anterior and just superior to the external auditory canal (Fig. 2a).

**Fig. 3:**
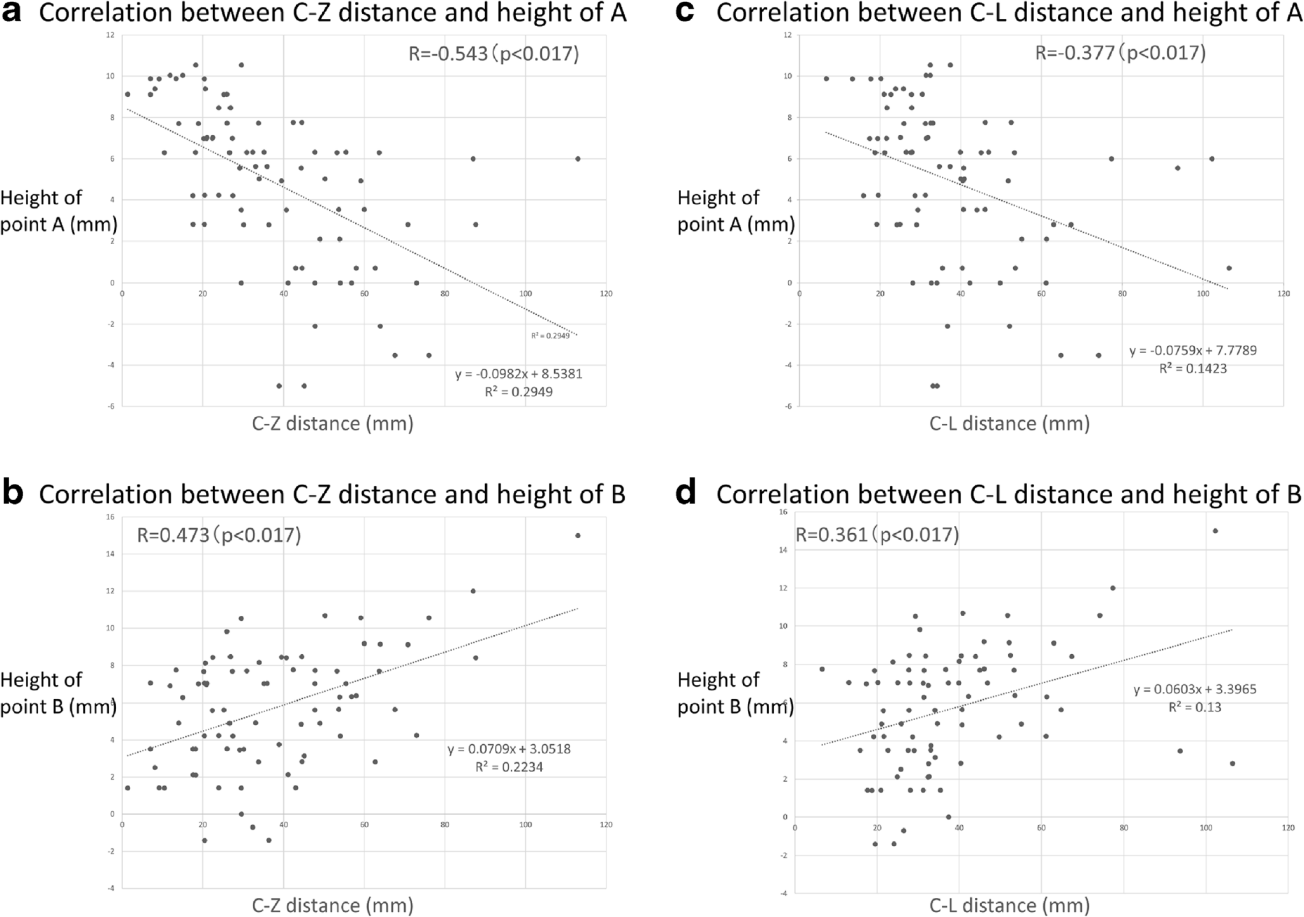
The C-Z distance, which relates inversely to the need for removal of the zygoma, is inversely correlated with the height of the apex of the BA from the dorsum sellae (*r* = 0.543, *p* < 0.017 after Bonferroni correction) (**a**) and positively correlated with the height of the bifurcation of the ICA (*r* = 0.473, *p* < 0.017 after Bonferroni correction) (**b**). The C-L distance, which relates inversely to the need for removal of the zygoma, is inversely correlated with the height of the apex of the BA from the dorsum sellae (*r* = 0.377, *p* < 0.017 after Bonferroni correction) (**c**) and positively correlated with the height of the bifurcation of the ICA (*r* = 0.361, *p* < 0.017 after Bonferroni correction) (**d**)

The measurements of points A and B are summarized in Table 1. The distances of point C from the zygomatic arch and lateral canthus were also calculated (Table 1). The C-Z distance, which relates inversely to the need for removal of the zygoma, was inversely correlated with the height of the apex of the BA from the dorsum sellae (*r* = −0.543, *p* < 0.017 after Bonferroni correction), (Fig. 3a) and was positively correlated with the height of the bifurcation of the ICA (*r* = 0.472, *p* < 0.017 after Bonferroni correction) (Fig. 3b).

**Table 1:** Pearson correlation coefficients of point C with primary parameters

Correlation		*r* value	*p* value
C-Z distance (mm)	Height of A (mm)	−0.461255428	*p* < 0.017
37.1 ± 21 (1.4–113)	4.9 ± 3.8 (−5–10.5)		
	Height of B (mm)	0.373358558	*p* < 0.017
	5.7 ± 3.1 (−1.4–15)		
	Width of B (mm)	−0.051929659	*p* = 0.608
	15.7 ± 3.4 (8.4–35.1)		
C-L distance (mm)	Height of A	0.331391373	*p* < 0.017
37.9 ± 18.9 (6.6–106.5)	Height of B	0.039853473	*p* < 0.017
	Width of B	0.167523808	*p* = 0.095

Height of A, the vertical height of the apex of the BA from the dorsum sellae; height of B, the vertical height of the bifurcation of the ICA from the dorsum sellae; width of B, the lateral breadth of the bifurcation of the ICA from the midline; C-Z interval, the distance of point C from the mid-zygomatic point (this inversely reflects the necessity of removal of the zygoma); C-L interval, the distance of point C from the lateral canthus (this inversely reflects the necessity of removal of the orbital rim)

*A *P* value lower than 0.017 after Bonferroni correction was considered significant

As well as the C-Z distance, the distance from point C to the lateral canthus (C-L distance), which corresponds to the need for removal of the orbital rim, had a significant correlation with the height of point A (*r* = −0.377, *p* < 0.017 after Bonferroni correction) (Fig. 3c) and point B (*r* = 0.361, *p* < 0.017 after Bonferroni correction) (Fig. 3d) (Table 1).

We also studied data from five patients with BX aneurysms who underwent surgery and reviewed the virtual images for preoperative simulation to verify the need for an OZA. The same items as those previously described were measured retrospectively.

From 2011 to 2014 at Nara Medical University, a total of 246 patients with cerebral aneurysm underwent surgical and endovascular treatment. Of these 246 patients, 17 had aneurysms that were located in the BX, including 10 patients who underwent endovascular treatment and 7 who underwent surgical treatment (2 via the subtemporal approach, 3 via the standard pterional approach, and 2 via the OZA). The five patients who underwent surgery via the standard pterional route and via the OZA were used to retrospectively review the surgical trajectory simulated by 3DCTA. Characteristics and operative procedures of these patients are listed in Table 2. Four patients underwent clipping of the aneurysm, and the remaining patient was treated with coating by the wrapping technique. The aneurysm of this patient (number 3) was surgically treated via the pterional approach [one aneurysm on the left middle cerebral artery (MCA) and one aneurysm on the BA-SCA]. A larger aneurysm in the left MCA was treated with neck clipping, and the BA-SCA aneurysm was approached via the standard pterional approach, which did not provide a sufficient operative field for neck clipping (Table 2).

**Table 2:** Measured values in five patients with BX aneurysms treated surgically

No.	Age (years)/ Sex	Location /size	Ruptured/ Unruptured	Height of A (mm)	Height of B (mm)	Width of B (mm)	C-Z distance (mm)	C-L distance (mm)	Approach
1	58 M	Basilar tip /7 mm	Ruptured	3	6	16.8	87	85.1	Clipping via right pterional
2	77 F	BA-SCA /5 mm	Ruptured	6	9	13.9	57	47.9	Clipping via left pterional with anterior clinoidectomy
3	74 F	BA-SCA /5 mm	Unruptured	9	12	9	36	31	Coating via left pterional
4	79 F	Basilar tip /3 mm	Ruptured	12	6	16.8	15	29.5	Clipping via right OZA
5	36 F	BA-SCA /7 mm	Unruptured	4	2.8	18	23.3	28.7	Clipping via right OZA

BA-SCA, basilar artery-superior cerebellar artery; A, apex of the basilar artery; B, bifurcation of the internal carotid artery; Z, mid zygomatic point; L, lateral canthus; OZA, orbitozygomatic approach

The height of the BX or point A ranged from 3 to 12 mm. Although patient no. 5 with the highest BX was operated on via the OZA, another patient undergoing the OZA had a BA-SCA aneurysm within 4 mm of the apex. Patients operated on via the OZA had the bifurcation of the ICA or point B of less than 6 mm in height, and the patient operated on without the OZA had point B of more than 6 mm in height.

**Fig. 4:**
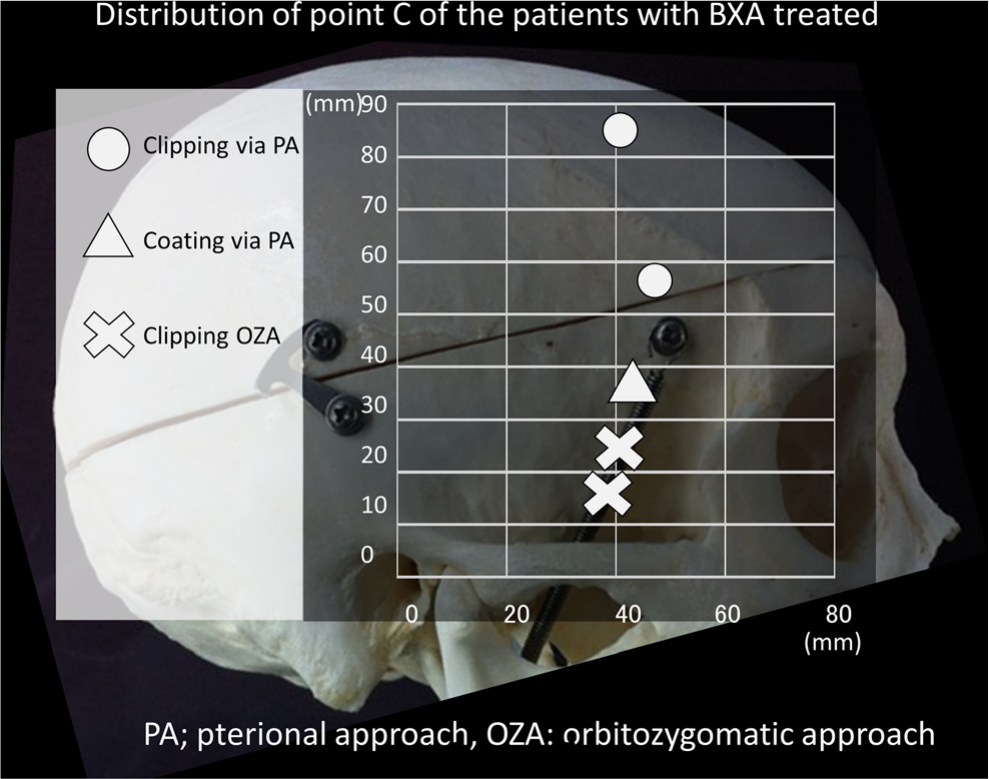
Point C in five patients with BX aneurysms treated surgically is plotted on the image of the skull. Two aneurysms in which the C-Z intervals were less than 3 cm (*white cross*) were approached via the OZA. Among the three patients who did not require the OZA, the C-Z intervals were more than 5 cm for two patients in whom neck clipping was accomplished (*white circle*). In the remaining patient treated via the standard pterional approach, clipping of the aneurysm could not be accomplished (*white triangle*)

The C-Z and C-L distances of these five patients ranged widely (Fig. 4). Clipping without the OZA was possible in patients 1 and 2, in whom the C-Z and C-L distances were more than 4 cm. There were two patients with BX aneurysm treated with clipping via the OZA in whom the C-Z and C-L distances were less than 3 cm. In patient number 3, the C-Z and C-L distances were 3.6 and 3.1 cm, respectively, which meant that the OZA may provide the appropriate operative field for clipping the aneurysm.

## Representative case

**Fig. 5:**
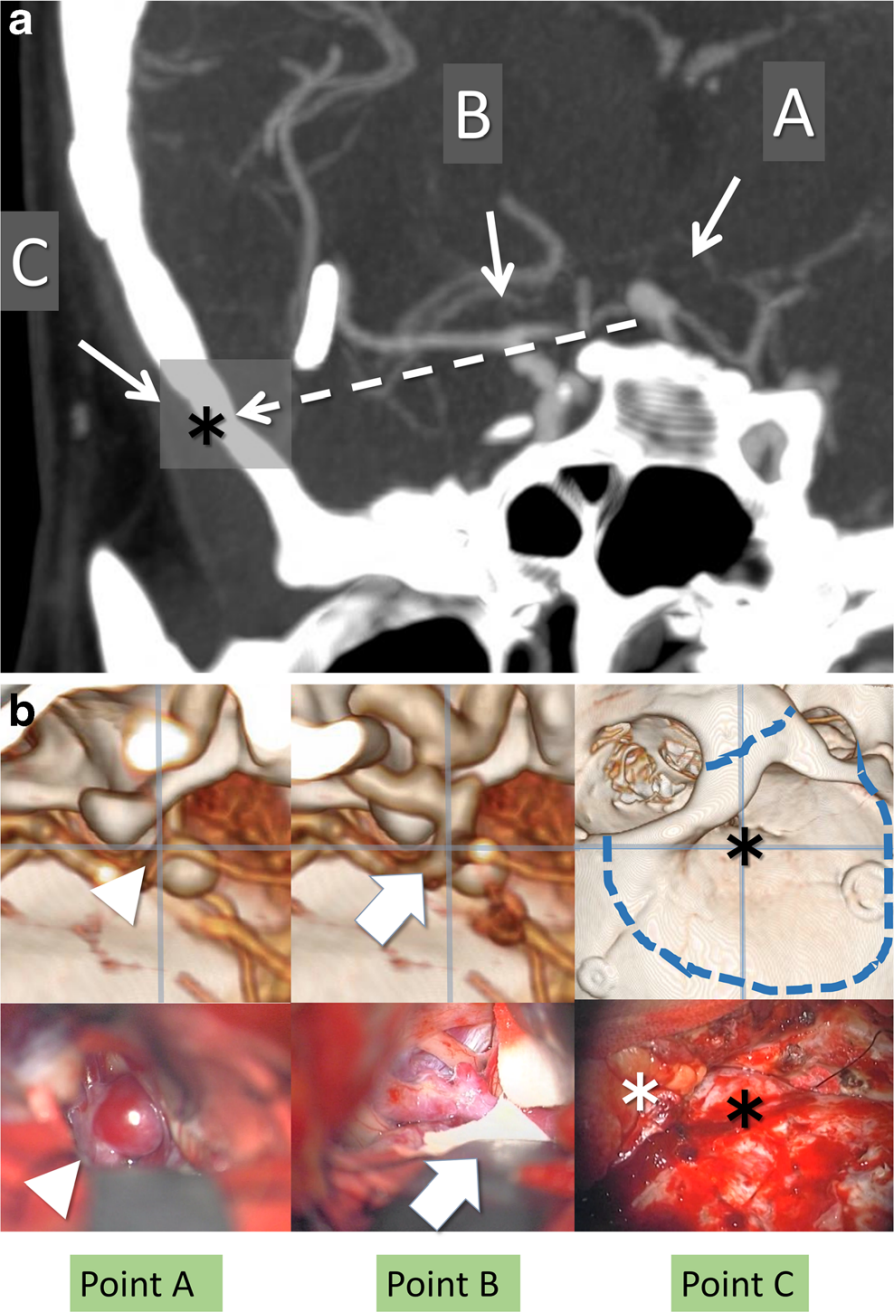
A 36-year-old female with a history of subarachnoid hemorrhage due to a ruptured middle cerebral artery aneurysm on the right side presented with de novo formation of an aneurysm in the BA-SCA with rightward projection. The images of the simulation by 3DCTA show the trajectory through the bifurcation of the ICA, with point C measured at 2.3 cm above the zygomatic arch and 2.9 cm from the lateral canthus. The 3D images along the trajectory of the ABC line demonstrate the spatial relationship between the aneurysm and the distal part of the ICA indicating the necessity of the OZA (**a**). Upper figures show 3D images along the simulated trajectory on each point from A through C. Lower figures show the intraoperative view in real surgery relevant to the points from A through C. The 3DCTA of patients after surgery shows obliteration of the aneurysms with multiple clips via the OZA (**b**)

A 36-year-old female with a history of subarachnoid hemorrhage (SAH) because of a ruptured MCA aneurysm on the right side had been treated with direct clipping 7 years earlier. Follow-up magnetic resonance angiography (MRA) demonstrated de novo formation of an aneurysm in the BA-SCA with rightward projection. The aneurysm was 7 mm in diameter, and treatment was indicated to reduce the risk of SAH recurrence. The tortuosity of her vertebral artery was remarkable, and the aneurysm projected laterally and had a relatively wide neck. Direct clipping was chosen instead of coil embolization based on these anatomical features, as well as her young age. The apex of the BA was located just above the level of the dorsum sellae, but the distal portion of the ICA on the right side was short, and the level of the bifurcation of the ICA was low-set from the dorsum sellae. The images of the simulation by 3DCTA showed that the trajectory from the bifurcation of the ICA to the aneurysm was at a lower angle than that estimated according to the height of the BX. Point C was measured 2.3 cm above the zygomatic arch and 2.9 cm from the lateral canthus (Fig. 5a).

We chose the right OZA to use the retrocarotid space to access the dome of the aneurysm. After orbitozygomatic osteotomy, the right temporal lobe was retracted posteriorly by dissecting the Sylvian fissure as widely as possible. The posterior temporal artery was detached from the medial surface of the temporal lobe, and the arachnoid membrane that was adhered to the uncus of the temporal lobe and oculomotor nerve was dissected sharply. Then, Liliequist’s membrane was opened to enter the interpeduncular cistern. The intracranial ICA was low-set, as estimated preoperatively, and upward viewing from below provided by the OZA was useful for observing the apex of the BA around the aneurysm while gently retracting the posterolateral surface of the ICA with a spatula. Inspection and dissection of perforators surrounding the aneurysm allowed us to apply a straight clip to the neck, leading to complete obliteration of the aneurysm (Fig. 5b). The patient’s postoperative course was uneventful without any sequelae.

## Discussion

A wide variety of approaches for BX aneurysms has been reported previously, including the pterional approach, the subtemporal approach, the translaminar terminalis approach, and the OZA [5, 7, 15, 16, 19]. Selection of the OZA for BX aneurysms is often dependent on the height of the BX from the dorsum sellae. However, the approach to BX aneurysms is sometimes dependent on the position of the intracranial portion of the ICA, because the ICA can limit the trajectory to a BX aneurysm [9]. Many studies have described how to mobilize the ICA to establish a wider operative field [8, 11, 18]. Even though mobilization of the ICA can be done by a variety of methods, such as anterior clinoidectomy, dissection of the distal dural ring, and division of the posterior communicating artery (PcoA), it can be obtained only in the proximal part of the intracranial ICA [3, 10, 18]. When a short ICA blocks the access route to a BX aneurysm, the OZA has been reported to be helpful [9]. In fact, the spatial relationship between the ICA and the BA is thought to be a very important point to consider with regard to the surgical approach. However, definitive standards have not yet been established.

This is the first study to quantify the correlation between the locations of the BA and the ICA via 3DCTA and the need for the OZA. The simulations were then retrospectively applied to patients who had undergone operative management of BX aneurysms to validate the utility of these data in selecting the surgical approach. The 3D virtual simulation showed the importance of the height of the ICA and BA when considering the surgical trajectory to access a BX aneurysm. Conventional angiography can provide powerful spatial resolution for depiction of the intracranial vasculature. Three-dimensional rotatory digital subtraction angiography can show a virtual image that can be manipulated as the reconstructed images from any angle [6] . However, catheter angiography is limited in that the posterior circulation is not depicted at the same time as the anterior circulation. Indeed, the bony structure was not visualized in conjunction with the imaging of vasculature, which represents a disadvantage when we simulate the surgical procedure in advance. Virtual images obtained from 3DCTA can provide multiple types of information on the same screen, which can help surgeons simulate the real operation.

In addition to the BA and ICA, the posterior clinoid process is a structure of paramount importance that often blocks the proximal control of the BA during the microscopic approach to BX aneurysms [5, 20]. The spatial relationship between the vascular structure and the clinoid process is well visualized as three-dimensional virtual images, which could be helpful and useful for surgeons. In this study, we did not address low-set basilar aneurysms that should be treated via the subtemporal or transpetrosal approaches [7, 13]. While considering such approaches, we should pay attention to the posterior clinoid process, as well as to the venous structures, such as the vein of Labbe, the superior petrosal sinus, or the inferior temporal vein, which can be evaluated by multimodal simulations, including MR venography and dynamic CTA [2, 12]. Even though the bony and vascular structures are well described with 3DCTA, the brain parenchyma and cranial nerves cannot be visualized on the same screen. Hybrid techniques using multiple modalities including MR imaging, SPECT, and ultrasound have been developed for preoperative simulation in many types of surgeries, including those used to treat neurovascular compression disease [1, 14, 17]. Further investigation will yield more information in the field of aneurysmal surgery.

The AB line and the resulting point C are important references when considering the surgical approach for BX aneurysms. There are many studies describing mobilization of the ICA to widen the operative field. Quantitative analysis showed evidence of widening and enlargement of the operative field. However, most procedures, including anterior clinoidectomy, dural ring dissection, optic canal unroofing, and even division of the PcoA, can mobilize the ICA at the C2 portion. In fact, the area between the optic nerve and the ICA can be widened by mobilization of the ICA. The retrocarotid window, or the area between the mobilized ICA and the oculomotor nerve, can also have an enlarged width. However, the terminal portion of the ICA arises from the ACA, MCA, and many perforators. The proximal parts of the ACA and MCA also have many perforators, which are immobile because of connections with the brain parenchyma by perforators themselves and by the strict arachnoid network. Namely, the operative field is limited in the vertical direction, because the bifurcation of the ICA is anchored. Therefore, if the ICA controlling the operative field does not provide sufficient visualization of the BX, it is necessary to change the angle of the trajectory, for which an orbitozygomatic osteotomy would be added or another approach would be chosen. Simulation images made from 3DCTA are also just one of the references to use when planning the surgical approach. Therefore, we should determine a comprehensive strategy to access the aneurysm according to the patient’s clinical characteristics and other neuroradiological examination modalities.

Although this study included a small number of patients with BX aneurysms treated surgically after preoperative simulation using 3DCTA, we advocate that the OZA should not only be considered based on the individual heights of the BA and the ICA, but also on the spatial relationship between the BA and the ICA corresponding to point C. If point C is very low and near the orbital rim, or there are short C-Z and C-L distances, the OZA should be used to access a BX aneurysm. Inversely, if both C-Z and C-L distances are more than 4 cm, we may be able to access the apex of the BA without the need for the OZA.

## Pitfalls and limitations of 3DCTA simulation

When using 3DCTA simulation, we should consider the invisible structures on the images made from CT scans, such as the oculomotor nerve, tentorium, and brain parenchyma, which are all important components that can limit the operative field. In this study, we randomly sampled 3DCTA data from 80 patients with various lesions, including cerebrovascular disease, tumors, and traumatic conditions. Among these enrolled patients, there were no patients with BX aneurysms. The number of patients with BX aneurysms undergoing real surgery is very small. Therefore, the tendencies and findings that were seen in this 3D simulation study cannot be generalized to patients with BX aneurysms. A variety of factors influence the creation of the operative field, such as swelling of the brain parenchyma or the thickness of the temporal muscle [4]. Therefore, an examination is necessary in each individual case. In real aneurysmal surgery, especially for BX aneurysms, not only the approach, but also the characteristics of the aneurysm are extremely important for clipping. They include the size of the aneurysm and its neck, the shape and direction of the dome, atherosclerotic change of the wall of the aneurysm, and surrounding perforators, which strongly affect the difficulty of clipping. In this study, it was not possible to evaluate these factors in BX aneurysmal surgery. We should always consider all factors, including the trajectory and the characteristics of the aneurysms, in each individual case.

## Conclusions

While imaging the virtual trajectory of the BX through the ICA, the height of the BX and the height of the bifurcation of the ICA were significantly correlated with the need for an OZA. However, the correlations of the heights of the ICA and BX with the need for OZA were not very strong individually. It was necessary to determine the spatial relationship between the BA and the ICA in order to decide whether an OZA is needed in real surgery. We proposed the point C, projected on the cranium by the extended line connecting BX (point A) with the bifurcation of ICA (point B) made by data from 3DCTA, as a potentially useful reference to determine the need for an OZA when accessing BX aneurysms.
